# Leaf senescence induced by EGY1 defection was partially restored by glucose in *Arabidopsis thaliana*

**DOI:** 10.1186/s40529-016-0120-3

**Published:** 2016-02-03

**Authors:** Cuiyun Chen, Jin Wang, Xin Zhao

**Affiliations:** Stress Physiology and Ecology Laboratory, Cold and Arid Regions Environment and Engineering Research Institute, Chinese Academy of Sciences, Lanzhou, 730000 China

**Keywords:** *egy1*, Leaf senescence, Glucose, Senescence-associated genes

## Abstract

**Background:**

Ethylene-dependent gravitropism-deficient and yellow-green 1 (EGY1) protein is required for chloroplast development and photosynthesis conduction. The *egy1* deletion mutants have a yellow-green phenotype and reduced granal thylakoids. Furthermore, the yellow-green phenotype of *egy1* mutants is more obvious than that of wild-type (WT) plants with increasing leaf age, suggesting an early senescence in the *egy1* mutants. However, the relationship between EGY1 functions and leaf senescence still remains poorly understood.

**Results:**

We observed that *egy1* mutant leaves were more yellow than those of WT (the same age) in *Arabidopsis* *thaliana*. In accompany with this phenotype, leaf survival, chlorophyll content, *Fv/Fm* and soluble protein content decreased, and ion leakage increased significantly in *egy1* mutants compared to WT plants. At molecular level, the expressions of senescence-associated genes increased, and photosynthesis genes decreased significantly in the mutants compared to those in WT plants. Furthermore, after darkness treatment, the yellow-green phenotype of *egy1* mutants was more obvious than that of WT. These results indicate that the loss-of-function of *egy1* gene induces leaf senescence in *A. thaliana*. In addition, our results showed that the yellow-green phenotype, chlorophyll content and ion leakage of *egy1* mutants was partially restored after exogenously applied glucose for 5 weeks. At the same time, the expression of *hexokinase 1* (*HXK1*) and/or *senescence*-*associated gene 12* (*SAG12*) in *egy1* mutants growing on 2 % glucose was lower than that in *egy1* mutants without glucose.

**Conclusion:**

*EGY1*-defection induced leaf senescence and this senescence was partially restored by glucose in *A. thaliana*.

## Background

The leaf is a specialized photosynthetic organ of plants, serving as the major site of producing energy and nutrients. Its development stages include initiation, growth, differentiation, maturation and senescence. Leaf senescence, the last stage of leaf development, plays a key role in plant survival and/or death. During leaf senescence, plant cells undergo orderly changes in structure, metabolism and gene expression (Buchanan-Wollaston et al. 2003; Guiboileau et al. 2010). Among these, the degradation processes of chlorophyll, proteins and lipids have been largely investigated (Hortensteiner and Feller 2002; Wiedemuth et al. 2005). Chlorophyll degradation typically starts at the leaf margins and progresses to the interior of the leaf blade (Kakani et al. 2004). Protein degradation is the most significant breakdown process that takes place during senescence (Buchanan-Wollaston et al. 2003). Other organelles, such as the liposome, also undergo biochemical changes as senescence proceeds (Hung and Kao 1998).

Leaf senescence is usually triggered by internal age-dependent factors, which include the expression changes of senescence-associated genes (SAGs), and the mutants of leaf death inducing genes, jasmonic acid-associated genes and other key genes (Castillo and Leon 2008; Guo et al. 2004). However, some external factors such as the alterations of nutrient, light and other environmental factors also initiate leaf senescence (Guiboileau et al. 2010; Ono et al. 2001). Of course, the defect of a gene associated with producing a nutrient (such as sugar) has the same effects as the external factors mentioned above (Loreti et al. 2008). Sugar signaling pathways are important regulation mechanisms for leaf senescence in plants (van Doorn 2008), which consists of glucose, sucrose, trehalose and other hexokinase-independent sugar signaling pathways (Smeekens et al. 2009; Xiao et al. 2000). Among these, the glucose signaling pathway is considered as one of the most important mechanism responsible for senescence and has been extensively studied (Balasubramanian et al. 2008). And hexokinase 1 (HXK1) acts as the direct glucose sensor mediating multiple functions in the glucose repression and glucose promotion of transcription and growth (Cho et al. 2006).

Nuclear mutations that cause albino or pale green phenotypes because of reduced levels of chlorophyll in the chloroplasts have been found frequently in higher plants (Bellaoui and Gruissem 2004; Vinti et al. 2005). These mutants usually have normal leaf anatomies, but typically show defects in chloroplast ultrastructure and composition (Aluru et al. 2007). The mutants of *egy1* (which coding for a protein named ethylene-dependent gravitropism-deficient and yellow-green 1, EGY1) are such plants of *Arabidopsis thaliana* because EGY1 protein is required for chloroplast development and photosynthesis conduction (Chen et al. 2005; Guo et al. 2008), and the yellow-green phenotype of *egy1* mutants is more obvious than that of wild-type (WT) plants with increasing leaf age. Although the photosynthesis characteristics of *egy1* mutants have been studied extensively, the relation between *egy1* mutation and leaf senescence is completely unknown. Since there was little attention given to color mutants and leaf senescence (Yoshida et al. 2002), we aim to explore the effects of *EGY1*-defection on leaf senescence and to further elaborate its potential mechanism in this paper.

## Methods

### Plant materials and growth conditions

The T-DNA insertion line SALK-134931 was obtained from the *Arabidopsis* Biological Resource Center (ABRC, Ohio State University), and plants homozygous for the *egy1* mutation (also known as *egy1*-*2* in Chen et al. 2005) were used for further analysis. Wild-type and the mutant plants were grown under 10-h-light/14-h-dark cycle with a photon flux density of 120 μmol m^−2^ s^−1^ at 22 °C. To ensure synchronized germination, the seeds were sown and then maintained in darkness for 48 h at 4 °C.

For dark treatment, 3-week-old seedlings of WT and *egy*1 mutant plants were placed in complete darkness for 3 days at 22 °C.

### Complementation of the *egy1* mutants

For complementation of the *egy1* mutants, cDNA containing the EGY1 coding region was amplified by PCR with the sense primer 5′-GGATCCAATGGGGACTCTCAC-CAG-3′ and antisense primer 5′-CGAGCTCTCACTAGTGTACATACATGGC-3′. The PCR product was cleaved with BamHI and SacI and cloned into the plant expression vector pSN1301 under the control of the cauliflower mosaic virus 35S promoter. The construct was transformed into *Agrobacterium tumefaciens* strain C58 and introduced into *egy1* plants by the floral dip method (Clough and Bent 1998). Transformed plants were selected on MS medium (Murashige and Skoog basal liquid medium; Sigma-Aldrich) containing 50 mg mL^−1^ hygromycin. The success of the complementation procedure was confirmed by PCR analysis and chlorophyll contents detection on the resulting plants.

### Measurement of leaf survival, chlorophyll content, photochemical efficiency, soluble protein and ion leakage

All these parameters were measured with the fifth rosette leaves, which were harvested at specified days counted from the day of leaf emergence. Leaf survival was determined by the mortality curves, which was the percentage of leaves that didn’t show full yellow (full yellow leaf was considered as dead) versus all leaves. Chlorophyll contents were quantified as described by Sims and Gamon (2002).

The photochemical efficiency of PSII was deduced from chlorophyll fluorescence parameters measured using a portable plant efficiency analyzer (Hansatech Instruments, Morfolk, England). The ratio of maximum variable fluorescence to maximum yield of fluorescence (*Fv*/*Fm*) was used as a measure of the photochemical efficiency of PSII.

Protein was extracted in 10 mM Hepes–KOH (pH 8.0), 10 mM MgCl_2_, 330 mM sorbitol, 2 mM PMSF and concentration was determined with a protein assay kit (Bio-Rad, CA, USA) using bovine serum albumin as a standard. Membrane ion leakage was determined by measuring electrolytes leaked using a digital conductivity.

### Nucleic acid preparation and analysis

Total RNA was extracted from 100 mg of fresh tissues using TRIzol reagent (Invitrogen). For the determination of the gene’s expression, RT-PCR was performed using the following primers: sense (5′-TCGCTTTTGCCGCTGCCGTTAACATTAG-3′) and antisense (5′-AAAGCTAACACGAGCACCACCGCGAGG-3′). To ensure equal amount of RNA in each sample, RT-PCR analysis of *actin* cDNA was performed using the following primers: sense (5′-AACTGGGATGATATGGAGAA-3′) and antisense (5′-CCTCCAATCCAGACACTGTA-3′).

Northern blot analyses were performed essentially as described previously (Sambrook and Russell 2001). The fifth leaves were harvested from WT and *egy1* plants at 26, 33 and 40 days after germination, and RNAs were separated on a 1.0 % agarose/formaldehyde gel, then transferred onto Nylon membranes (Amersham Pharmacia Biotech). The membranes were probed with ^32^P-labelled cDNA probes specific for *senescence*-*associated gene 12* (*SAG12*), *SAG24*, *endo*-*xyloglucan transferase/xyloglucan endo*-*1,4*-*beta*-*d*-*glucanase* (*SEN4*), *chlorophyll a/b*-*binding protein* (*CAB*), and *the rubisco small subunit gene* (*RBCS*). Ethidium bromide staining was used as a loading control. Following high-stringency hybridization and washing, all the blots were exposed to X-ray film.

### Exogenous glucose treatment

*Arabidopsis thaliana* seeds were sown on half-strength MS plates containing various concentrations of glucose, and plates were placed at 4 °C for 2 days for vernalization. They were germinated and maintained at 22 °C in darkness for 6 days and illuminated at 22 °C for 12 h. The hypocotyl lengths of seedlings were measured.

At the same time, WT and *egy1* plants were grown on 2 % glucose + MS medium in a 500 cubic cm glass jar for 5 weeks, and then the phenotype were observed, the chlorophyll contents, ion leakage were determined, and the expressions of *hexokinase 1* (*HXK1*) *and senescence*-*associated gene 12* (*SAG12*) were quantified.

### Quantitative PCR Analysis

Primer pairs for the quantitative PCR were: 5′-GCAGACTTCTCTGTCCTCTGG-TAG-3′ (forward) and 5′-TCCAACAACATCTTGTCCAACTGC-3′ (reverse) for *HXK1*; 5′-AAGGAGGAAAACAATCGCTAC-3′ (forward) and 5′-GCAAACTGA-TTTACCGCAAG-3′ (reverse) for *SAG12*; 5′-CGTACAACCGGTATTGTGCT-GG-3′ (forward) and 5′-CTCTCTCTGTAAGGATCTTCATG-3′ (reverse) for *actin*. The quantitative PCR was performed with a Mx3000P Real-Time PCR System (Stratagene, Agilent, USA) using SYBR Green SuperMix (Takara) with the following conditions: 30 s at 95 °C; 40 cycles of 10 s at 95 °C, 10 s at 55 °C and 12 s at 72 °C, with final melting for 15 s at 65 °C. Melting curve analysis was performed to confirm the specificity of the amplification and to identify putative unspecific products. *Actin* mRNA, set to 100 %, was used as an internal standard in all experiments. The quantitative PCR experiments were repeated at least three times for a cDNA prepared from three batches of plants.

## Results

### Isolation of *egy1* mutants

**Fig. 1:**
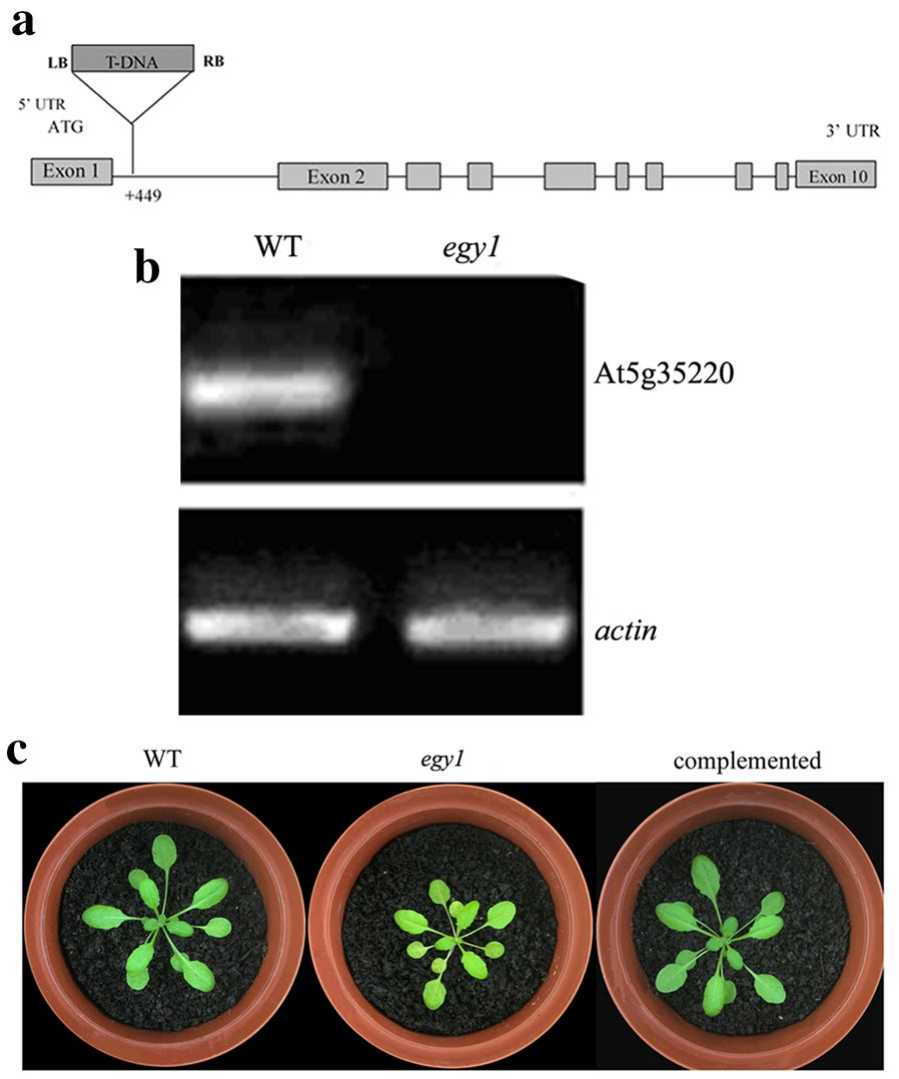
Expression of EGY1 and phenotypes of *egy1* mutants. Gene structure of EGY1 and location of T-DNA insert (**a**), RT-PCR analysis of EGY1 expression in wild-type and *egy1* leaves (**b**), phenotypes of wild-type, *egy1* and *egy1* complemented lines at 28 days after germination (**c**)

The T-DNA insertion line (SALK-134931, Fig. 1a) (Columbia background) was obtained from the *Arabidopsis* Biological Resource Center (ABRC) at Ohio State University. The homozygosity of plants was confirmed by PCR amplifying and sequencing. To study the effect of the T-DNA insertion on At5g35220 expression, we analyzed the results of RT-PCR, which showed that this gene was undetectable in the mutant, while it was normal expressed in WT plants (Fig. 1b). The oldest *egy1* mutant leaves had the most intense yellow-green phenotype (Fig. 1c). Complementation of *egy1* mutants was performed to ensure the function of EGY1 (Fig. 1c).

### Yellowing of *egy1* rosette leaves

**Fig. 2:**
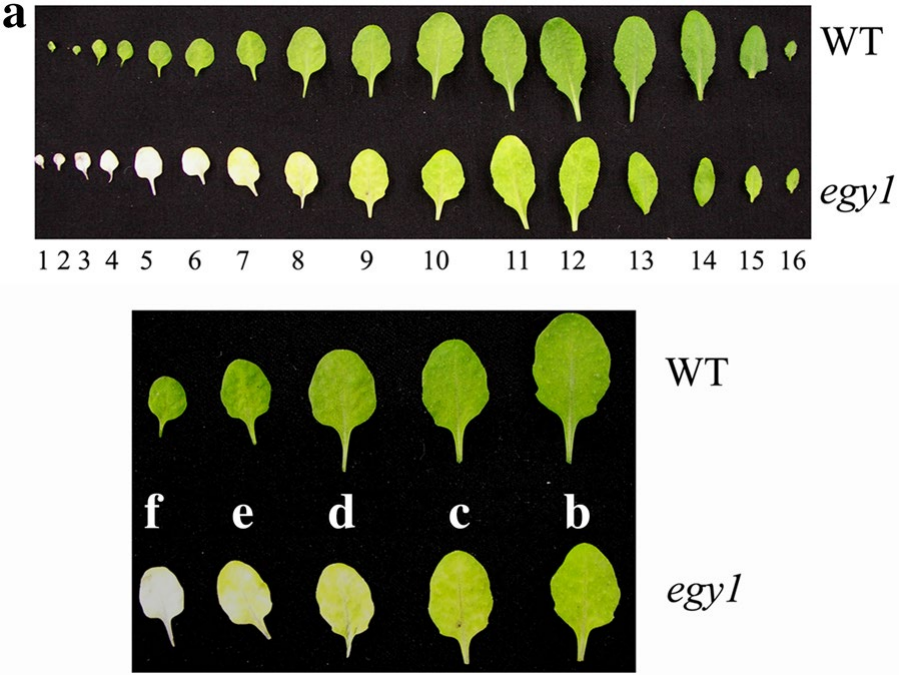
Phenotypes of *egy1* mutants in age-dependent senescence. A photograph of wild-type and *egy1* plants (**a**), visible senescence phenotypes in the *egy1* leaves (**b**–**f**), leaves were numbered from bottom to top. Chlorosis often initiates from midrib (**c**), and sometimes chlorotic lesions are formed (**d**) in *egy1* leaves. Chlorotic area expands from tip to base on an *egy1* leaf (**e**, **f**)

The fifth rosette leaves emerged on the same day both in the *egy1* mutants and WT plants. The yellow-green of young leaves and albino of old leaves in *egy1* mutant (Fig. 2a) was not due to difference in the number of leaves. Yellowing of the *egy1* leaves happened first at the tip of the leaf blades, later spread to the end of leaf blades. The leaves sometimes developed chlorosis along the midrib (Fig. 2b–f).

### Physiological changes in *egy1* leaves

In order to examine the physiological state of the *egy1* mutants, the survival, chlorophyll content, *Fv/Fm*, soluble protein content and ion leakage of the fifth leaves were measured in this study.

**Fig. 3:**
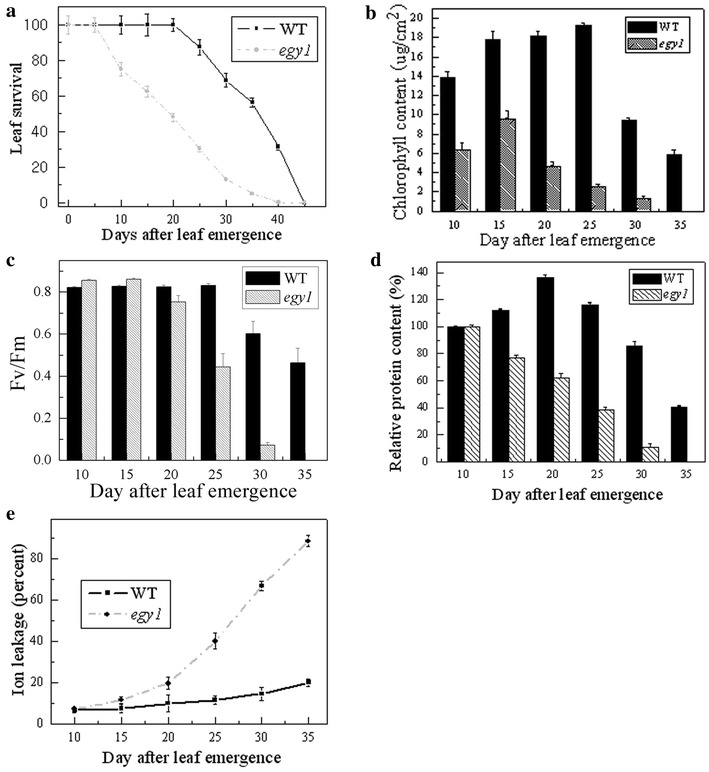
A time-course of changes in senescence indicators of the fifth leaves at indicated times after leaf emergence. Leaf survival (**a**), chlorophyll contents per leaf area (**b**), *Fv*/*Fm* (**c**), soluble protein contents per leaf (**d**) and ion leakage (**e**). Soluble protein contents were shown as relative values of those measured at 10 days. At 35 days, chlorophyll contents and soluble protein contents were undetected in the *egy1* mutants. The standard deviation was calculated from twelve samples for leaf survival; six for chlorophyll and *Fv*/*Fm*, and three for soluble protein and ion leakage measurements

We first examined the effect of visual leaf longevity (representing the leaf survival) in the *egy1* mutation (Fig. 3a). We observed that the leaf longevity of *egy1* was shortened by 40.1 %. The time it took 50 % of leaf population to survive was 22 days after leaf emergence in the mutants, while it was longer, 37 days, in the WT plants (Fig. 3a).

The chlorophyll content of *egy1* mutants at 10 days of leaf emergence was <50 % of that in the WT plants (Fig. 3b). Chlorophyll content decreased in *egy1* mutants after 20 days, while it was stable until 30 days of leaf emergence in WT leaves (Fig. 3b).

The *Fv*/*Fm* ratio, which reflects the photochemical quantum efficiency of PSII, was similar in *egy1* mutants and WT plants at 15 days of leaf emergence. But after 20 days, the *Fv*/*Fm* ratio began to decrease in *egy1* leaves, whereas it did not decline until 25 days in WT leaves (Fig. 3c).

At 10 days after leaf emergence, soluble protein contents were measured in *egy1* mutants and WT plants. Soluble protein began to decrease on the 15 days of leaf emergence in *egy1* mutant, while it increased until 20 days in WT leaves. Protein contents decreased below 60 % of the initial level at 25 days of leaf emergence in *egy1* mutants and after 35 days in WT (Fig. 3d).

Ion leakage, an indicator for the intactness of the plasma membrane, began to increase 15 days after leaf emergence in *egy1* leaves, and its ratio continued increasing significantly throughout the growth period. However, ion leakage in WT leaves began to increase until 30 days. Moreover, there was no increase in the ratio throughout the growth period in WT plants, unlike *egy1* mutants (Fig. 3e).

### Transcription of senescence-related genes in the *egy1* mutants

**Fig. 4:**
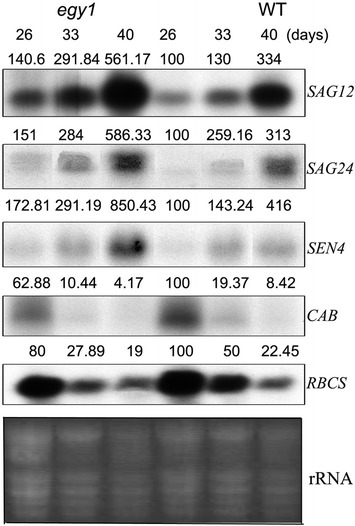
Transcript accumulation of senescence-related genes and genes related to photosynthesis in naturally senescing *egy1* leaves. The percentages of RNA levels shown above the lanes were estimated by comparison with levels found in wild-type corresponding samples taken at 26 days

To evaluate leaf senescence in the *egy1* mutants at molecular level, the transcription levels of *SAG12*, *SAG24*, *SEN4*, *CAB* and *RBCS* were examined by RNA gel-blot analysis. The transcripts of *SAG12* in *egy1* plants accumulated ca. 207 % at 33 days and ca. 400 % at 40 days compared with that of 26 days after germination, which was more than those in WT plants (Fig. 4). The transcripts of *SAG24* and *SEN4* were also increased in *egy1* mutants. Similarly, the transcript levels of *CAB* and *RBCS* decreased more obviously in *egy1* plants than those in WT plants with increasing leaf age (Fig. 4).

### The influence of darkness treatment

**Fig. 5:**
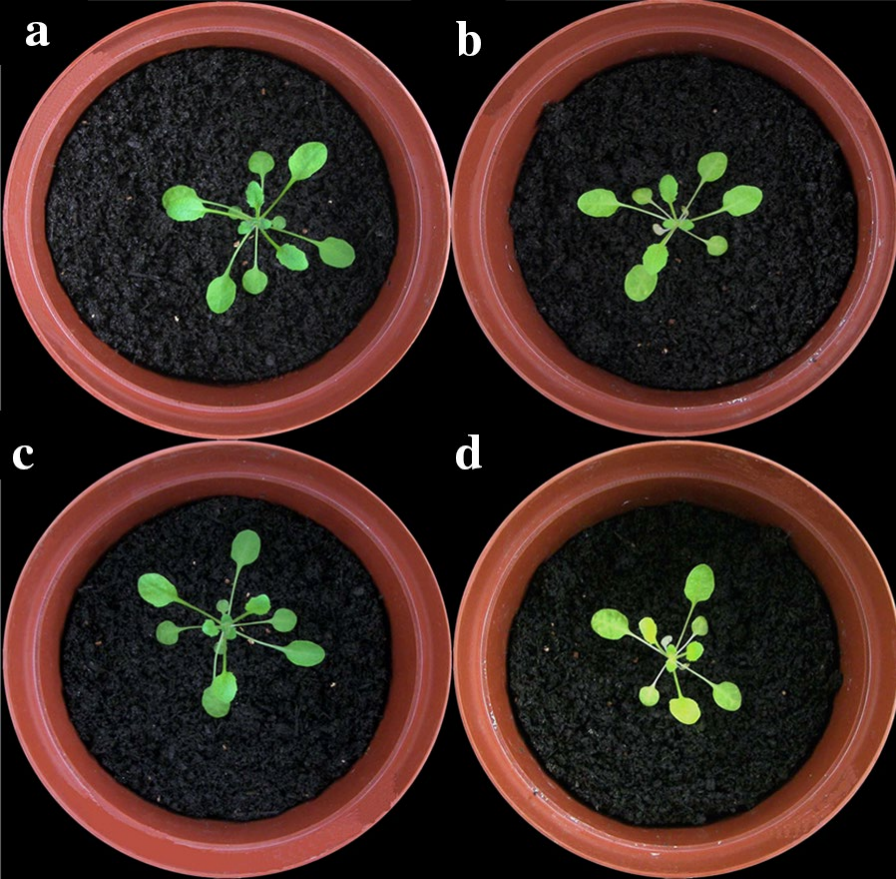
Yellowing of *egy1* leaves during dark treatment. Wild-type (**a**, **c**) and *egy1* (**b**, **d**) plants before dark treatment (**a**, **b**) and after 3 days (**c**, **d**) of dark treatment are shown

27-Day-old WT and *egy1* plants (Fig. 5a, b) were placed in a dark chamber in order to observe phenotypic changes due to darkness. After 3 days, the yellow-green phenotype was more obvious in *egy1* mutants (Fig. 5d) than that in WT plants (Fig. 5c).

### Leaf senescence of *egy1* mutants was delayed by exogenously applied glucose

**Fig. 6:**
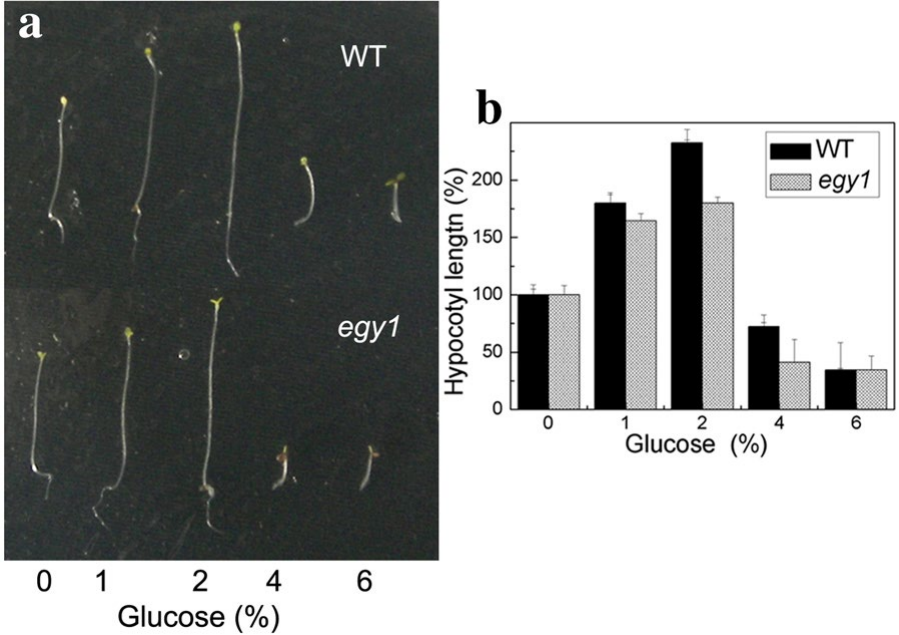
Sugar response in the *egy1* seedlings. Wild-type and *egy1* seedlings were grown on a medium containing various concentrations of glucose for 6 days under darkness and then for 12 h under illumination. Phenotypes of the seedlings (**a**), hypocotyl length of seedlings was measured and standard deviations were calculated from 12 seedlings (**b**)

**Fig. 7:**
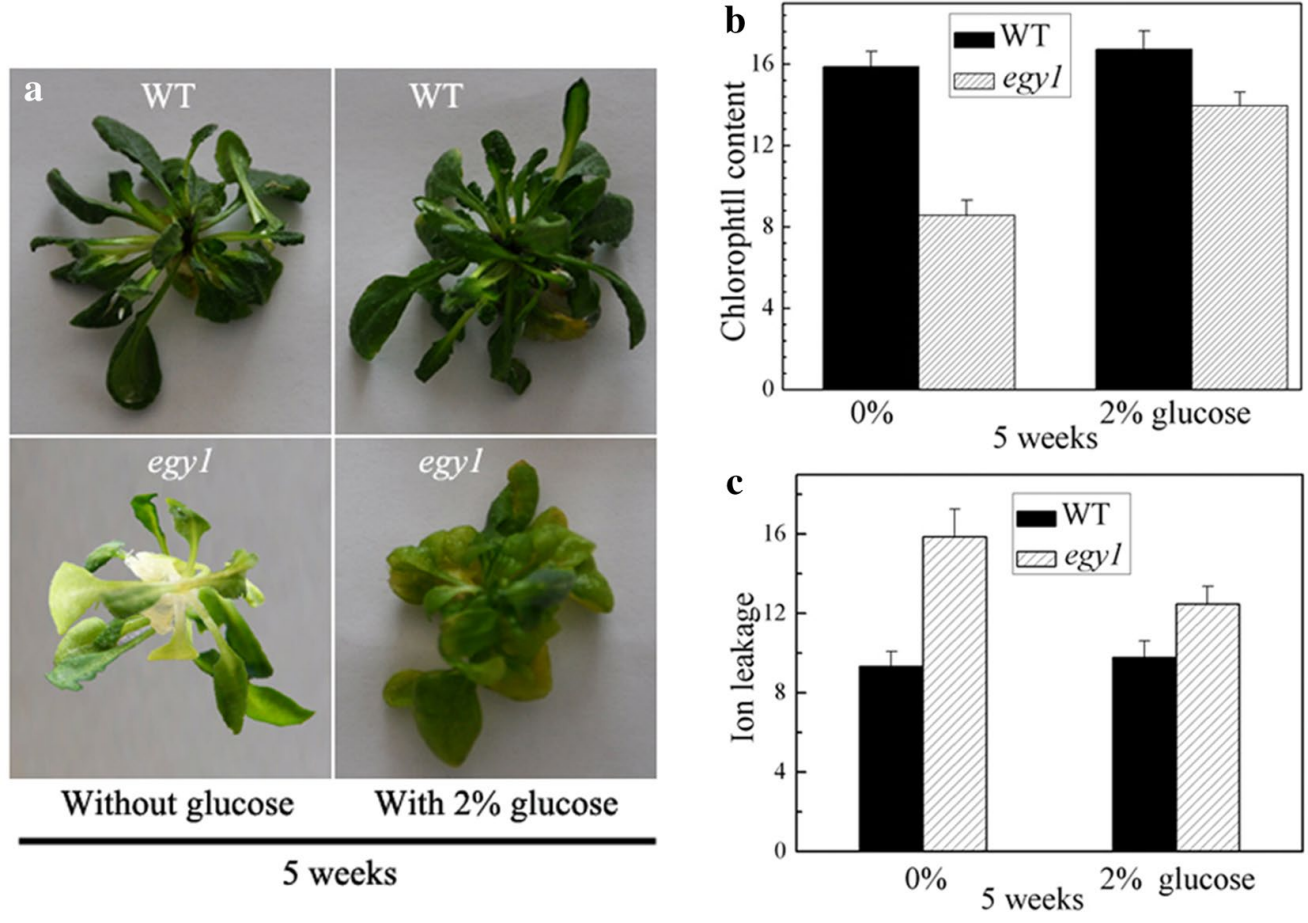
Phenotypes (**a**), chlorophyll contents (**b**) and ion leakage (**c**) in wild-type and *egy1* plants supplied with 2 % glucose for 5 weeks

Cotyledon greening in WT was almost the same as in *egy1* mutants regardless of treatment with or without 1 % (56 mM) and 2 % (111 mM) glucose (Fig. 6a). However, cotyledons greening was inhibited in *egy1* plants treated with 4 % (222 mM) and 6 % (333 mM) glucose, and in WT plants with 6 % glucose (Fig. 6a). Moreover, hypocotyl elongation was increased from 0 to 2 % glucose both in WT and *egy1* seedlings (Fig. 6b), while it was inhibited under 4 % and 6 % glucose both in *egy1* and WT seedlings (Fig. 6b). Next we planted WT and *egy1* mutants on 2 % glucose for 5 weeks. The results showed that yellow-green phenotype of *egy1* mutants was partially restored by glucose treatment (Fig. 7a). Chlorophyll contents were increased and ion leakage was decreased in *egy1* mutants applied with 2 % glucose compared to those without glucose treatment (Fig. 7b, c).

**Fig. 8:**
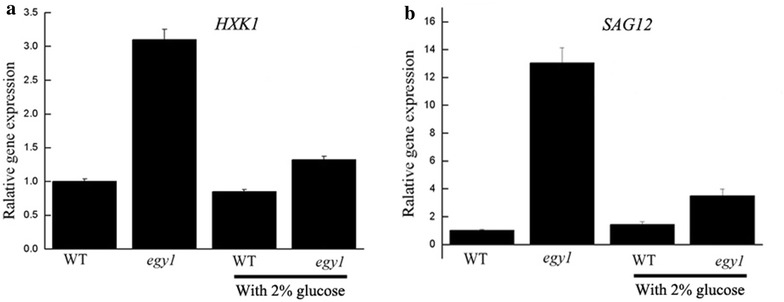
The relative transcription levels of *hexokinase 1* (*HXK1*) (**a**) and *senescence*-*associated gene 12* (*SAG12*) (**b**) in *egy1* and wild-type plants growing on 0 % and 2 % glucose. The transcripts were determined by real-time PCR and normalized against *actin*. The transcription levels are relative to the WT set at 0 % glucose. The quantitative PCR experiments were repeated at least three times for a cDNA prepared from three batches of plants

The results from quantitative PCR analysis indicated that the high expressions of *HXK1* and *SAG12* in *egy1* mutants were impaired by 2 % glucose treatment for 5 weeks (Fig. 8).

## Discussion

In general, leaf senescence is dependent on age and developmental stage under normal conditions and in the absence of external stress, and the leaf yellowing during senescence is usually observed in old leaves. In this study, the leaf yellowing phenotype of *egy1* mutant and WT plants is also related to the increasing leaf age and leaf normal development. However, *egy1* mutant leaves are more yellow than those of WT plants at the same age and development stage (Figs. 1, 2), suggesting that *egy1* mutants have an early-senescence phenotype.

To further confirm the early-senescence phenotype, we measured the physiological characteristics associated with senescence in *egy1* mutants. The leaf longevity was lower in *egy1* mutants than that of WT plants (Fig. 3). Similar results were detected in *apg7*-*1* mutants of *Arabidopsi*s which showed an early-senescence phenotype (Doelling et al. 2002). Chlorophyll can provide basic information of photosynthesis and photosynthesis defects is an important feature of senescence. Our results showed that chlorophyll contents were also reduced in *egy1* mutants (Fig. 3), which was similar to the reported data by Frick et al. (2003) in *porB*-*1porC*-*1* double mutant of older seedlings and by Stettler et al. (2009) in *mex1* leaves. Proteins are fundamental components of all living cells and are necessary for the proper functioning of an organism, and could be decreased during leaf senescence. We found that *egy1* mutation had decreased soluble protein contents (Fig. 3), similar tendency was also detected in *hys1* and *apg7*-*1* early-senescence mutants (Doelling et al. 2002; Yoshida et al. 2002). Ion leakage is an indicator of membrane integrity. When leaf senescence, membrane became fragile and leak. In *egy1* mutants, ion leakage was increased (Fig. 3). Taken together, these results suggested that the *egy1* mutants had early-senescence traits.

Leaf senescence is accompanied by increased expression of *senescence*-*associated genes* (*SAGs*) and decreased expression of genes related to photosynthesis (Lim et al. 2003; Zentgraf et al. 2004). The transcript levels of *SAG12*, *SAG24* and *SEN4* genes were increased in senescent leaves (Wu et al. 2008; Yoshida et al. 2001), while *CAB* and *RBCS* genes were down-regulated during leaf senescence (Woo et al. 2001). Similar results were found both in *egy1* mutants and in WT plants with increasing leaf age. It is notable that, *SAG12*, *SAG24*, *SEN4* increased and *CAB* and *RBCS* decreased more obviously in *egy1* mutants compared to WT plants (Fig. 4). These results indicated that leaf senescence occurred indeed in transcript level in *egy1* plants.

Leaf senescence could be induced by external stresses, such as darkness. Darkness treatment induced uniform and rapid senescence (Chrost et al. 2004; del Rio et al. 2003; Mishev et al. 2009). After 3 days of dark treatment, *egy1* mutant leaves were more yellow while the phenotype of WT plants had no obvious changes (Fig. 5), suggesting that *egy1* mutants had an early-senescence phenotype under normal conditions and in darkness treatment. So EGY1 should have an important role in leaf normal development in *A. thaliana*. As the *egy1* mutants have a wide variety of senescence symptoms, the *egy1* gene most likely functions as a regulatory component controlling functional leaf senescence rather than as a protein directly executing the senescence process (Kim et al. 2009; Lim et al. 2010).

The chloroplasts in *egy1* mutants had reduced granal thylakoids, poorly developed lamellae networks and decreased photosynthetic proteins (Chen et al. 2005). The loss in photosynthesis caused cellular sugar starvation, and lack of energy (Patro et al. 2014). Sugar starvation is known to affect leaf senescence, repress hypocotyl elongation, cotyledon greening and shoot development (Arru et al. 2008; Gazzarrini and McCourt 2001). In tobacco plants, sugar starvation accelerated leaf senescence, suggesting that sugar signaling could directly or indirectly promote leaf senescence (Wingler and Roitsch 2008). This starvation could be restored after exogenously applied sugars through sugar metabolism with complex sugar signaling network. Yoshida et al. (2002) found that *hys1* seedlings with exogenously applied sugars were hyper-responsive to glucose, implying that sugar may play a role in the early induction of senescence. In our study, hypocotyl elongation was increased in *egy1* seedlings growing on 2 % glucose. Furthermore, the yellow-green phenotype of *egy1* mutants was partially restored by 2 % glucose (Fig. 7), suggesting that the symptom of early senescence was relieved after glucose supplementation. Meanwhile, the expressions of *HXK1* and *SAG12* (which are usually at a high level in senescence leaves) were obviously decreased in *egy1* mutants by 2 % glucose (Fig. 8), implying that the early senescence of *egy1* mutants may be due to sugar starvation. It therefore could be restored by exogenous glucose treatment. With regard to the inhibition of cotyledons greening or hypocotyl elongation by 4% or 6 % glucose, it is a result from the effects of high concentrations of glucose in the medium and has been reported in the previous study (Jang et al. 1997).

## Conclusion

In conclusion, our results suggest that leaf senescence induced by *EGY1*-defection may be due to sugar starvation and can be partially restored by glucose in *A. thaliana*. EGY1 regulated leaf senescence provides key information to understand the molecular mechanism of leave senescence in plants.

## Abbreviations

EGY1: ethylene-dependent gravitropism-deficient and yellow-green 1
SAG: senescence-associated gene
CAB: chlorophyll a/b-binding protein
RBCS: the rubisco small subunit gene
SEN4: endo-xyloglucan transferase/xyloglucan endo-1,4-beta-d-glucanase
